# The Accuracy of Ultrasonography in Detection of Ulnar Collateral Ligament of Thumb Injuries; a Cross-Sectional Study

**Published:** 2018-02-19

**Authors:** Babak Shekarchi, Mohammadreza Mokhdanzadeh Dashti, Mostafa Shahrezaei, Ebrahim Karimi

**Affiliations:** 1 Radiology Department, Imam Reza Hospital, AJA University of Medical Sciences, Faculty of Medicine, Tehran, Iran.; 2 Emergency Department, Be’sat Hospital, AJA University of Medical Sciences, Faculty of Medicine, Tehran, Iran.; 3 Orthopedics Department, Imam Reza Hospital, AJA University of Medical Sciences, Faculty of Medicine, Tehran, Iran.

**Keywords:** Ultrasonography, collateral ligament, ulnar, injuries, magnetic resonance imaging, dimensional measurement accuracy

## Abstract

**Introduction:**

Timely diagnosis and treatment of traumatic injury to ulnar collateral ligament (UCL) of thumb is of special importance for preserving the full function of the hand. Therefore, the present study has been designed with the aim of evaluating the accuracy of ultrasonography in detection of these injuries.

**Methods:**

The present diagnostic accuracy study was performed on trauma patients over 15 years old who had clinical evidence of injury to UCL of thumb and were admitted to the emergency department. All patients were evaluated regarding injury to the mentioned ligament via ultrasonography and MRI and finally, the accuracy of ultrasonography in this regard was measured considering MRI as the reference test.

**Results:**

20 individuals with the mean age of 38.60 ± 13.45 (16 – 64) years were evaluated (60% male). Based on ultrasonography and MRI findings 7 (35%) individuals and 7 (35%), respectively had complete ligament rupture (kappa: 0.560 (95% CI: 0.179 – 0.942)). Sensitivity, specificity, positive and negative predictive value, and positive and negative likelihood ratio of ultrasonography in detecting injuries of the mentioned ligament were 71.42 (30.25 – 94.88), 84.61 (53.66 – 97.28), 71.42 (30.25 – 94.88), 84.61 (53.66 – 97.28), 2.5 (0.71 – 8.82), and 0.18 (0.04 – 0.67), respectively.

**Conclusion:**

Based on the findings of the present study, performance of ultrasonography by a radiologist in the emergency department has 80% accuracy in detecting traumatic injuries of UCL of the thumb.

## Introduction:

Ulnar collateral ligament (UCL) of thumb is one of the major protective ligaments of metacarpophalangeal joint (1). Damage to this ligament usually happens following hyper-abduction or hyper-extension of the mentioned joint (2, 3). This injury is among the most common injuries of thumb with a frequency of 50 cases in 100000 population each year, following accidents and trauma and sports injuries (2, 4-7). Rupture of this ligament leads to decrease in the ability of the patient to hold objects and make a fist using the thumb and therefore, timely diagnosis and treatment of this injury is very important for preserving the function of the hand (8, 9).

Diagnosis of injury to this ligament is mostly based on clinical examination and inability to bear pressure on the thumb in the valgus position (10). Plain radiography, ultrasonography, and magnetic resonance imaging (MRI) are available imaging tools for evaluating UCL injuries of thumb. Plain radiography shows fractures properly and ultrasonography and MRI are good for showing the details of injury to soft tissue (11-13). Sensitivity and specificity of MRI in detection of UCL of thumb have been estimated as 100% in a study by Harper et al. (14). However, the accuracy of ultrasonography in detection of this type of injuries has varied from 40% to 92% between various studies (15-18). Since ultrasonography has become an available and inexpensive tool in most emergency departments, there is an increasing inclination towards using ultrasonography in detection of various problems in patients. Therefore, the present study has been designed with the aim of evaluating the accuracy of ultrasonography in detection of traumatic injuries of UCL of thumb.

## Methods:

***Study design and setting***

The present diagnostic accuracy study was performed on trauma patients who had clinical evidence of injury to UCL of thumb and were admitted to the emergency department of Be’sat Hospital, Tehran, Iran, over a period of 2 years. All the patients were evaluated regarding injury to the mentioned ligament via ultrasonography and then MRI and finally, the accuracy of ultrasonography in this regard was measured considering MRI as the reference test. Protocol of the present study was approved by the ethics committee of AJA University of Medical Sciences and all researchers adhered to the principles of Helsinki Declaration. Before inclusion in the study, informed written consent for participation in the study was obtained from the patients or their companions.

***Participants***

Participants of the present study consisted of patients over 15 years of age who had clinical evidence of traumatic injury to UCL of metacarpophalangeal joint of the thumb that were included via consecutive non-probability sampling. Those with non-traumatic injuries of UCL, visible fracture and rupture at the anatomic site under evaluation, and also those who did not cooperate in performing imaging were excluded from the study. No sex limitation was considered in the present study.

***Imaging***

Ultrasonography of tendon was performed by a skilled radiologist using GE voluson E8 bt10 device and 12MHz linear probe of MSK system. In addition, MRI of the tendon was done using a 1.5 tesla device and from T1 view for anatomical interpretation and T2 view for pathological interpretation. Injuries were reported as complete rupture, without rupture, and/or with minor rupture. The radiologist interpreting MRI and the sonologist were not aware of each other’s findings and the clinical findings of the patient.

***Data gathering***

Data gathering was done by a senior emergency medicine resident using a pre-designed checklist including demographic data of the patients (age, sex, height, and weight) as well as ultrasonography and MRI findings regarding injury of UCL.

In this study presence of tenderness at the time of performing Valgus Stress test and also Valgus Stress range more than 30 degrees (15-30 = normal) were considered as clinical evidence of probable injury of UCL (19).

***Statistical analysis***

Minimum sample size required for the present study, considering 15% prevalence of UCL injury (20), 95% confidence interval, 0.17 precision and considering 15% probability of loss samples was estimated as 20 cases. Data underwent statistical analysis using SPSS 21 statistical software. Data were presented as frequency (%) or mean ± standard deviation. For calculating sensitivity, specificity, positive and negative predictive value, positive and negative likelihood ratio, and accuracy of ultrasonography in detection of traumatic injuries of UCL with 95% confidence interval, Vassarstats medical calculator was used. In this study, MRI was considered as the reference test. 

For evaluating the agreement rate between the findings of ultrasonography and MRI, calculation of kappa coefficient with 95% confidence interval was applied. In this study, kappa coefficient less than 0.20 was considered as little agreement, 0.21 – 0.40 as poor, 0.41 – 0.60 as average, 0.61 – 0.80 as good and 0.81 – 1.00 as very good.

## Results:

20 individuals with the mean age of 38.60 ± 13.45 (16 – 64) years were evaluated (60% male). Table 1 depicts the baseline characteristics of the studied patients. Most patients were in the 30 – 45 years age group (45%). Mean body mass index (BMI) of the patients was 22.95 ± 3.02.

Based on ultrasonography findings 7 (35%) individuals had complete rupture of UCL of thumb and others had minor ruptures or no rupture at all. Based on MRI findings, these rates were 7 (35%) individuals with complete‌ rupture, and 13 (65%) patients without or with minor injuries. Agreement rate between the findings of ultrasonography and MRI based on calculation of kappa coefficient was 0.560 (95% CI: 0.179 – 0.942). Table 2 shows the screening performance characteristics of ultrasonography in detecting injuries of UCL of thumb in comparison with MRI. The overall accuracy of ultrasonography in the mentioned field was estimated as 0.80 (95%CI: 65.34 – 94.27).

## Discussion:

Based on the results of the present study, performance of ultrasonography by a radiologist in the emergency department has 80% accuracy in detecting traumatic injuries of UCL of the thumb. Rate of agreement between the findings of ultrasonography and MRI in this regard was estimated as average. It seems than considering its relatively low sensitivity, ultrasonography cannot yet be used as a proper screening tool in detecting injuries of UCL of thumb.

**Table 1:** Baseline characteristics of the studied patients

Variable	**Values **
**Sex **	
Male	12 (60)
Female	8 (40)
**Age (year)**	
< 30	5 (25)
30 – 45	9 (45)
≥ 45	6 (30)
**Body mass index**	
18.5 – 25	12 (60)
≥ 25	8 (40)
**Height (m)**	1.76 ± 0.07
**Weight (kg)**	77.5 ± 11.9

Data are presented as mean ± standard deviation or frequency and percentage.

**Table 2:** Screening performance characteristics of ultrasonography in detection of traumatic injuries of ulnar collateral ligament of the thumb in comparison with MRI

**Characteristics **	**Values (95% CI)**
**True positive**	5
**False positive**	2
**True negative**	11
**False negative**	2
**Sensitivity **	71.42 (30.25 – 94.88)
**Specificity **	84.61 (53.66 – 97.28)
**Positive predictive value **	71.42 (30.25 – 94.88)
**Negative predictive value**	84.61 (53.66 – 97.28)
**Positive likelihood ratio**	2.5 (0.71 – 8.82)
**Negative likelihood ratio**	0.18 (0.04 – 0.67)

Sensitivity and specificity of MRI in detecting injuries of UCL of thumb in various studies have been estimated as 96% to 100%, and 100%, respectively and it is currently considered as the standard test in detection of these injuries (14, 15, 21). Of course, detection of tendon injuries using MRI has its own complications and requires skill of the interpreter. Despite ultrasonography being available, portable, and costing less, it also depends on the operator and therefore, its accuracy varies widely and has been reported as 40% to 92% in different studies (17, 18, 22). 

In a study by Melville et al. in 2013 sensitivity and specificity of ultrasonography method were estimated to be about 100% (23). In contrast, Papandrea et al. in 2008 estimated sensitivity and specificity of ultrasonography to be 76% and 81%, respectively. In addition, the positive and negative predictive values of ultrasonography were estimated to be 74% and 87%, respectively, in the mentioned study (16). The results of the present study are in agreement with the studies performed in this regard to a great extent.

In this study, attempts were made to eliminate limitations such as training and insufficient skill of the operator by asking a radiologist to perform the ultrasonography. But despite the performance of ultrasonography by a radiologist, the number of false positive and false negative cases or in other words incorrect report was relatively significant (4 cases out of 20 reports or 20%).

The first studies carried out in the field of assessing the diagnostic accuracy of ultrasonography in detecting ULC of thumb injuries belong to 1980 to 2000. Maybe the reason for ultrasonography not being known as a proper method for widespread use in this regard is that first, the mentioned problem is not a life-threatening emergency and suspected cases in clinical examination will not undergo further interventions and following fixation, will be visited by an orthopedic specialist or a surgeon with delay. Second, since a radiologist is not available at all times of the day, even if the accuracy of this method is high it will not be available 24 hours a day. In addition, if emergency medicine specialists gain the required skill in this regard by undergoing special trainings, surgeons and orthopedic specialists will not rely on it for planning surgery and will apply more accurate methods such as MRI. Therefore, despite the numerous advantages of bedside ultrasonography in the emergency department, we should be cautious about its type of application and exaggeration of its abilities. The wide confidence interval of likelihood ratio of ultrasonography in detection of the mentioned tendon’s injuries challenges it as a proper screening test. It seems that in the presence of a low-risk and accurate method such as MRI, performing ultrasonography cannot be of much help in management of these patients and accurate planning regarding their need for repair. 

## Limitations:

Small sample size might be the most important limitation in generalizing the findings. In this study by blinding the sonologist and MRI interpreter to the clinical data, we tried to prevent any bias in this regard.

## Conclusion:

Based on the findings of the present study, performance of ultrasonography by a radiologist in the emergency department has 80% accuracy in detecting traumatic injuries of UCL of the thumb. Rate of agreement between the findings of ultrasonography and MRI in this regard was estimated as average. It seems than considering its relatively low sensitivity, ultrasonography cannot yet be used as a proper screening tool in detecting injuries of UCL of thumb.
